# Advertisements for children’s entertainment products in a popular parenting magazine: sedentary or active?

**DOI:** 10.15171/hpp.2017.09

**Published:** 2016-12-18

**Authors:** Corey H. Basch, Aleksandar Kecojevic, Valerie Cadorett, Charles E. Basch

**Affiliations:** ^1^Department of Public Health, William Paterson University, Wayne, NJ 07470, USA; ^2^Health and Behavior Studies, Teachers College, Columbia University, NY, NY 10027, USA

**Keywords:** Children, Sedentary, Entertainment products, Advertisements

## Abstract

**Background:** The purpose of this study was to describe advertisements of children’s entertainment products in a popular magazine, Parents, and to determine if they illustrated behavior that was physically active or sedentary.

**Methods:** The sample was comprised of Parents magazines (January 2010 to December 2015). Coding involved determining if the advertisement was promoting sedentary or active behavior.

**Results:** Nearly all of the 169 advertisements in the sample (n = 166; 97.6%) were for products that depicted sedentary behavior. The most common types of entertainment products advertised were DVDs (n = 72), plastic stacking products (n = 18), books (n=14), and electronic devices (n = 13). The most popular theme that appeared in the advertisements was the entertainment product would enhance intelligence (n = 85; 50.3%, 95% CI: 0.43-0.58). The overwhelming majority (n = 136; 80.5%. 95% CI: 0.76-0.87) of the advertisements involved the presence of a character.

**Conclusion:** This type of advertising does not contribute to the nation’s goals of increasing physical activity among youth.

## Introduction

Recent research indicates that, in the United States, there are nearly 13 million obese children and adolescents (ages 2 to 19 years old).^1^ There is a correlation between sedentary behaviors and childhood obesity, which may be affected by the intake of foods with low nutritional value, by a dearth of physical activity (which can depend on the types of products they seek for entertainment), and increased contact with advertisements for foods of low nutritional quality.^2^

In the United States, $21.18 billion was generated by retail sales of children’s entertainment products in 2011.^3^ Billions of dollars per year are spent marketing a wide array of products to children, a portion of which are for entertainment.^4^ Advertisements are used to captivate children’s attention, produce a positive feeling toward the product, and serve as memory triggers for products.^5^

Advertisers portray affection and sensitive children in ads to elicit an emotional response from consumers, creating the illusion of a tight bond between parents and children.^6^ As evidenced by marketing research of food advertising directed at children and adolescents, parents could be manipulated into purchasing products that could have adverse effects on their children’s health.^7^ While entertainment products, such as puzzles and arts and crafts, stimulate creative thinking, they often promote sedentary behavior, which is not recommended for optimal health.^8^ Growing technology, such as handheld video games and electronic media, have been contributing to the extended amount of time children spend being sedentary.^9^ For example, on an average school day, over 40% of high school students in America reportedly spend 3 or more hours a day on computer or video games or used a computer for non-school work.^10^ These kinds of entertainment products and games are advertised in parents’ magazines.

Mass media, including magazines, shares both health promoting and health hindering messages to millions of people. In this study, we extend our prior research focusing on parenting magazines.^11-13^ Advertisements for children’s entertainment products in parenting magazines may influence parenting practices, contributing to development of physically active or sedentary behaviors of children. The purpose of this study was to describe the advertisements of children’s entertainment products in a popular parenting magazine,* Parents,* with respect to whether they depicted physically active or sedentary behavior. Our aim was to contribute to the emerging literature regarding the nature of children’s leisure activities.

## Materials and Methods

At the current time, *Parents* reaches an audience of more than 13 million readers, with a high social media presence, including one million Twitter followers.^14^ Our sample comprised of 6 years of *Parents* magazines (from January 2010 to December 2015), which totaled 72 issues. Advertisements promoting children’s entertainment products that could target caregivers, parents, and/or children were included. All children’s entertainment product advertisements taking up a quarter of a page or more were included. Advertisements that spanned multiple pages were only counted one time.

The coding sheet used in this study was based on a previous study analyzing advertisements in *Parents.*^12^ This particular study is ancillary to a larger study examining sedentary and active images of children in this magazine.^15^ One researcher, (V.C.) began coding by totaling the number of pages followed by examining the entertainment product advertisements presented throughout the magazine. The first step was to determine the category of the entertainment product. The entertainment product categories were created inductively and are listed in Table 1. The second step was to determine if the entertainment product in the given advertisement was promoting sedentary or active behavior. Active entertainment products were defined as promoting physically energetic behaviors. Examples include: climbing, entertainment products, and computerized exercise based games. Sedentary entertainment products were defined as promoting stationary behaviors. Examples include: board games, DVDs, books, CDs, video games (without a physical activity component), and plastic stacking entertainment products. To establish intra-rater reliability, a sample of 9 magazines were re-coded two months after the initial coding took place. A high level of agreement was indicated at 97.2%.

**Table 1:** Entertainment product categories and their description

**Toy Category**	**Description**
Adventure	The product promoted dangerous and an exhilarating experience. The toys lead to searching for things, jungles, journeys, solving mysteries, and venturing into unknown territory.
Enhance creativity	Products that allowed children the freedom to build, create, and develop.
Excitement	The children in the advertisement appeared to be excited as a result of playing with the product or waiting their turn to play with the toy. Children may have appeared anxious or sitting at the edge of the seat. The advertisement may have contained the word "exciting."
Fun	The children in the ad appeared to be having fun as a result of playing with the product. The advertisement may have contained the word "fun."
Making you popular	The product would make other children want to play with the toy, leading to more friends. Children would want the product because someone they know has it.
Enhance intelligence	The product depicted in the ad would teach children something, such as addition, subtraction, letters, and words.
Empowerment	The advertisement portrayed making children mentally strong, independent, and successful as a result of playing with it.
Sleep aid	The product being advertised helped sooth the child leading to a better sleep.

Descriptive statistics, including frequencies and percentages, were calculated using the SPSS version 23.0. Studies that do not involve human subjects are considered exempt from human subjects review at William Paterson University and Teachers College, Columbia University.

## Results

The total of 169 children’s entertainment product advertisements were coded. The greatest number of advertisements was in 2013 (n=45) and declined in 2014 (n=7) and 2015 (n=12). Nearly all of the advertisements (n=166; 97.6%, 95% CI: 0.95-1.00) were entertainment products that depicted sedentary behavior. The most popular theme was that the product would enhance intelligence (n=85; 50.3%) and was fun (n=28, 16.6%). These categories were followed by adventure (n=14, 8.3%), excitement (n=14, 8.3%), and enhancing popularity (n=13, 7.7%). Less common themes were products that served as a sleep aid (n=9, 5.3%), led to empowerment (n=3, 1.8%), or creativity (n=3, 1.8%). The overwhelming majority (n=136; 80.5%) of the advertisements involved the presence of a character.

The most common types of entertainment products advertised were DVDs (n=72), plastic stacking products (n=18), books (n=14), and electronic devices (n=13). These categories were followed by video games/apps (n=11), stuffed animals (n=11), and dolls (n=9). Less commonly advertised entertainment products were walkers/jumpers (n=5), board games (n=4), CDs (n=3), climbing products (n=3), robots (n=2), bubbles (n=2), crafts (n=1), and wagons (n=1).

## Discussion

Child play with toys can aid in growth and development.^16^ When a child and their caregiver play together with toys, it can enhance early brain development.^16^ Our paper is the first to document whether children’s advertisements for entertainment products depict active or sedentary images in a popular parenting magazine. This data showed that nearly all of the advertisements in this sample of magazines depicted images that were sedentary. While the number of advertisements declined over time, the appearance of sedentary images in advertisements was consistently more prominent than physically active images. These findings were consistent with our prior study.^15^ In that study, we found that out of over 11,000 images of children, almost 90% depicted sedentary behavior.^15^

There are many factors that affect obesity, and media is just one. This study is limited in that it was cross sectional (although collecting data over a six-year time period), involved only one coder, included a specified number of years, and involved only a single publication that reaches a defined population of readers. Print media can be influential and to the extent that advertisements primarily portray children engaged in sedentary activities. This does not contribute to the nation’s goals of increasing the extent to which youth are more physically active. It is advised that toy advertisements claiming educational or developmental promotion should be scrutinized, especially if the advertisement claims enhancing intelligence.^16^ It is also advised that screen time should be limited and avoiding toys that promote violence and negative stereotypes.^16^ Future research is needed to examine decision-making processes of parents in purchasing entertainment products for children. Parents may need assistance in making informed decisions about purchasing entertainment products that promote children’s physical activity.^17^

## Ethical approval

The Institutional Review Boards at William Paterson University and Teachers College, Columbia University do not review studies, such as this, that do not include human subjects.

## Competing interests

The authors declare that they have no conflict of interest.

## Authors’ contributions

CHB and CEB conceptualized the study, designed data collection meth­odology, and led manuscript development. AK conducted data analysis and assisted with manuscript development. VC was responsible for data collection and assisted with manuscript development.

## Acknowledgments

None.
